# The gap in injury mortality rates between urban and rural residents of Hubei province, China

**DOI:** 10.1186/1471-2458-12-180

**Published:** 2012-03-12

**Authors:** Qing Liu, Lan Zhang, Junlin Li, Dan Zuo, Deguang Kong, Xingfu Shen, Yi Guo, Qingjun Zhang

**Affiliations:** 1 The State Key Laboratory of Virology(2004DA105204) and Department of Epidemiology and Health Statistics, School of Public Health, Wuhan University, 185# Donghu Rd.,, Wuhan 430071, China; 2 Hubei Province Centers for Disease Control and Prevention (CDC), 6# Zhuo-dao-quan-bei Rd., Wuhan 430079, China

## Abstract

**Background:**

Injury is a growing public health concern in China. Injury death rates are often higher in rural areas than in urban areas in general. The objective of this study is to compare the injury mortality rates in urban and rural residents in Hubei Province in central China by age, sex and mechanism of injury.

**Methods:**

Using data from the Disease Surveillance Points (DSP) system maintained by the Hubei Province Centers for Disease Control and Prevention (CDC) from 2006 to 2008, injury deaths were classified according to the International Classification of Disease-10^th ^Revision (ICD-10). Crude and age-adjusted annual mortality rates were calculated for rural and urban residents of Hubei Province.

**Results:**

The crude and age-adjusted injury death rates were significantly higher for rural residents than for urban residents (crude rate ratio 1.9, 95% confidence interval 1.8-2.0; adjusted rate ratio 2.4, 95% confidence interval 2.3-2.4). The age-adjusted injury death rate for males was 81.6/100,000 in rural areas compared with 37.0/100 000 in urban areas; for females, the respective rates were 57.9/100,000 and 22.4/100 000. Death rates for suicide (32.4 per 100 000 vs 3.9 per 100 000), traffic-related injuries (15.8 per 100 000 vs 9.5 per 100 000), drowning (6.9 per 100 000 vs 2.3 per 100 000) and crushing injuries (2.0 per 100 000 vs 0.7 per 100 000) were significantly higher in rural areas. Overall injury death rates were much higher in persons over 65 years, with significantly higher rates in rural residents compared with urban residents for suicide (279.8 per 100 000 vs 10.7 per 100 000), traffic-related injuries, and drownings in this age group. Death rates for falls, poisoning, and suffocation were similar in the two geographic groups.

**Conclusions:**

Rates of suicide, traffic-related injury deaths and drownings are demonstrably higher in rural compared with urban locations and should be targeted for injury prevention activity. There is a need for injury prevention policies targeted at elderly residents, especially with regard to suicide prevention in rural areas in Central China.

## Background

Worldwide, injuries represent a substantial public health issue, accounting for approximately 5 million deaths in 2000, a mortality rate of 83.7 per 100 000 population [1]. Injuries are an emerging public health problem in China that cause almost 700,000 - 750,000 deaths each year with a death rate of 65.2 per 100,000 population [2]. Injury is the fourth leading cause of death next to malignant tumors, cardiovascular diseases and diseases of respiratory system [3]. It is estimated that the annual number of deaths from injuries in China will reach 1,400,000 by 2010 and 2,500,000 by 2050.

China's rapid economic growth has been accompanied by regional inequality. The gross domestic product per person in the richest province is 13 times greater than that in the poorest province [4]. According to most estimates, mean per capita income in urban China is more than triple that in rural areas [5]. Urban populations, which account for only 30% of the total population, use 80% of total health resources [6]. The urban-rural disparity has been widely recognized as an important issue for injury control in China. Using 10 years of national Disease Surveillance Points (DSP) data to examine fatal injury in China, Yang et al. reported higher mortality rates among rural residents, with reported average annual age-adjusted death rates of 38.7 per 100,000 population for urban residents and 74.6 per 100,000 populations for residents of rural areas. Injury death rates in the central and western rural areas were higher than those in eastern rural areas [7]. Other studies both from national samples and local samples in China also show higher injury mortality rates in rural areas [3,8-12]. However, these studies are limited by the lack of details (gender, age group, and mechanism of injury etc.) on urban-rural disparities. Hu and colleagues reported differences in injury mortality by gender, age group, and mechanism between urban and rural residents [13], but the data used in this analysis were from the Chinese Vital Registration system which has better data quality in urban than in rural areas resulting in significant biases in the overall statistics [14].

Data from Hubei Province Disease Surveillance Points (DSP) system provides important information on the demographics and distribution of injury mortality in a population of 6 million, about 10% of the total population of the province. The DSP system is more geographically representative than the Chinese Vital Registration system because it uses multi-stage cluster probability sampling [14]. The focus of this study is to describe and compare the injury mortality rates in urban and rural residents from 2006 through 2008 in Hubei Province, central China, by age, sex and mechanism of injury. It was hypothesized that rural populations would have an overall increased risk of injury-related mortality and that mechanisms of injury would vary, based on geographic residence.

Hubei Province, located in the centre of China, has an area of 185,900 km^2 ^and a population of 60.7 million (approximately 46.5 million in rural areas in 2007), residing in 17 municipalities and 99 counties. The per capita gross domestic product (GDP) of the province is 27,000 yuan (approximately US $ 4150) and the difference in average annual income between rural and urban areas is about fourfold. Injuries are the fourth leading cause of death both in Hubei Province and in China. It is important that Hubei Province and China focus attention on this important public health issue and develop strategies to decrease the burden of injuries. Epidemiologic results from our study in Hubei Province will serve to inform the development of local and national injury prevention strategies in China.

## Methods

Death data from 2006 through 2008 were collected for this study through the Disease Surveillance Points (DSP) system, maintained by the Hubei Province Centers for Disease Control and Prevention (CDC) for a sample of about 10% of the total population of Hubei Province. Urban areas and rural areas were officially designated in 1955 with the introduction of nationwide state monopoly and rationing system for grain. Citizens living in the officially-designated urban areas and engaging in non-agricultural activities at that time were classified as non-agricultural *Hu Kou *population; others are agricultural *Hu Kou *population. In our study, the urban data from DSP system are mainly from the non-agricultural population of large and middle size cities, and the rural data are mainly from the agricultural population of counties. The primary sampling unit in urban areas was the city, and in rural areas, the county. Death data were collected using a stratified, multi-stage cluster probability sampling method. According to national socioeconomic classification criteria, an urban area was classified as a large, middle size, or small city based on population size. Rural areas were classified as class 1, class 2, or class 3 based on the health and economic situation in the area. The target sample size for each level was determined according to the proportion of the population resident at each level. A total of 3 districts (covered about 6.8% of its 0.98 million urban population) and 5 counties (covered about 11.8% of its 5.1 million rural population) were selected. All residents in selected area were monitored from 2006 through 2008. According to municipal and local regulations, death certificates were filled out and entered into the Cause of Death Reporting System by all the hospitals and community clinics in Hubei province. The district or county health officials oversaw and checked the daily reported deaths at the primary level, on a case-by-case basis. Non-hospital deaths were included in the Reporting System through verbal autopsy interviews by community clinicians on a door-to-door basis. About 50% and 80% of adult deaths occur at home in urban areas and in rural areas respectively. Even for those deaths that occur at home, there is often clinical evidence available from recent consultations with medical staff members at community or other hospitals. The standardised verbal autopsy procedure for the cause-of-death ascertainment in DSP system was described by Yang [14]. Diagnoses based on the verbal autopsy were found to be valid and reliable for most cause of disease both in urban and rural [15,16]. The district or county health officials were responsible for collecting and verifying these additional death certificates and adding them into the Reporting System on a weekly basis. The Hubei CDC carried out secondary data verification, investigation of missing reports, as well as technical training and support for staff involved in the reporting process.

Each death certificate recorded age, sex, and primary diagnosis of the cause of death. The cause of death was coded according to the International Classification of Disease-10^th ^Revision (ICD-10) [17]. Deaths with an underlying cause of death classified as "external cause of morbidity and mortality", including intentional self-harm (V01-Y98) were identified as injury deaths. The coding was conducted by trained medical personnel.

Crude annual injury mortality rates as well as gender- and age-adjusted rates per 100 000 population (and 95% CI) were calculated for Hubei Province. Population estimates from the 2000 Chinese Census were used to compute the denominators for injury mortality rates. The standard used to obtain adjusted rates was the sum of the populations by age and gender. Rate ratios (RRs) and 95% CIs were calculated for the rural and urban categories, using the urban category as the referent. The chi-square test was used for examining the urban-rural differences in injury mortality rates. When the assumptions for the chi-square test were violated, it was replaced by Fisher's exact test; *P *< 0.01 was selected as the statistically significant level.

Microsoft Excel and SPSS 11.0 statistical software were used for data management and analysis

Ethics approval for this research was obtained from the Medical Ethics Committee of Wuhan University.

## Results

**Figure 1:**
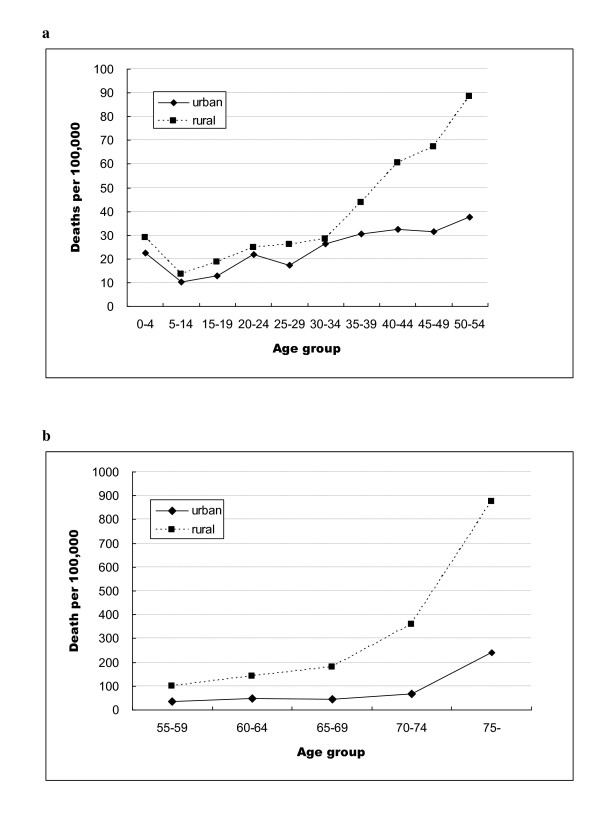
**Age-specific injury death rates in urban and rural residents, Hubei Province, 2006-2008: 1a: 0-54 year age group; 1b: ≥55 year age group**.

Crude and age-adjusted injury death rates for 2006-2008 were significantly higher in rural areas than in urban areas (Table 1). There was an approximate two-fold difference in the injury death rates for rural and urban areas in both males and females. Age-specific death rates for urban and rural residents are illustrated in Figure 1. Injury mortality rates increased starting at 15 years of age, leveled off at ages 15-34 years, but then dramatically increased among older populations. The highest injury death rates were seen among elderly residents of rural areas. It is noteworthy that the rural injury death rate (252.7 per 100 000, 95% CI: 245.4-259.9 per 100 000) for individuals over age 55 was 3 times the urban rate (83.2 per 100 000, 95% CI: 76.2-90.2 per 100 000).

**Table 1:** Average annual population, number of deaths, crude and age-adjusted rates per 100 000, and the rural-to-urban rate ratios, overall and by gender, 2006-2008

	No. of injury deaths, 2006-2008	Average annual population	Average annual crude rate per 100,000 (95%CI)	Crude rate ratio^a ^(95%CI)	Average annual age-adjusted rate per 100,000 (95%CI)	Adjusted rate ratio^a ^(95%CI)
**urban**	1174	1108126	35.3(34.6-38.8)	1.9^a^	30.1(28.3-32.0)	2.4^a^
**rural**	8540	4277771	66.6(66.3-69.1)	1.8-2.0	70.7(69.2-72.2)	2.3-2.4
**males**						
urban	724	536619	45.0(41.2-48.2)	1.8^a^	37.0(34.1-39.8)	2.2^a^
rural	5327	2177553	81.5(79.4-83.7)	1.7-2.0	81.6(79.4-83.8)	2.2-2.2
**females**						
urban	450	530354	28.3(25.7-30.9)	1.9^a^	22.4(20.1-24.6)	2.6^a^
rural	3213	2026408	52.9(51.0-54.7)	1.7-2.1	58.0(55.9-60.0)	2.6-2.6

^a ^Significant rate ratios at the 1% level

**Figure 2:**
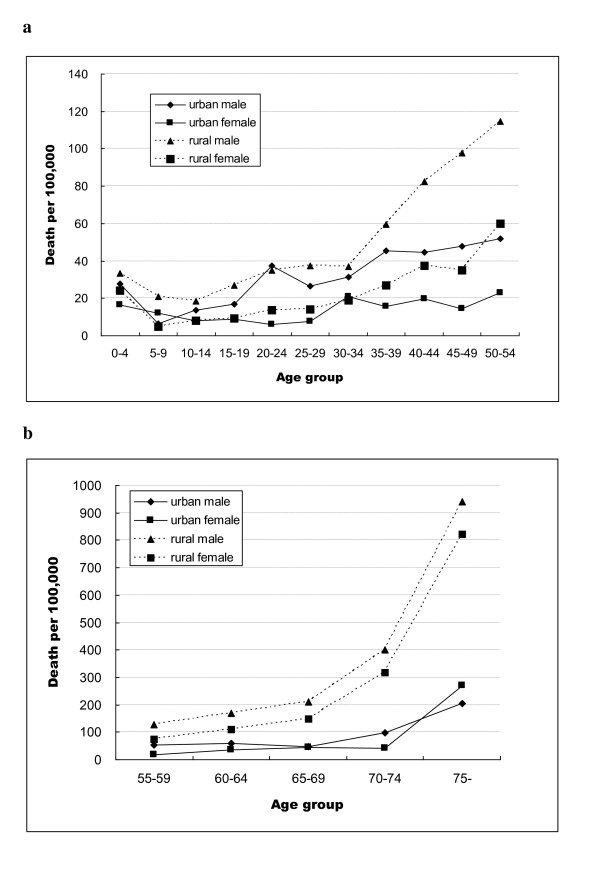
**Age and gender-specific injury death rates, urban and rural areas in Hubei Province, 2006-2008: 2a: 0-54 year age group; 2b: ≥55 year age group**.

Age- and gender-specific injury death rates are shown in Figure 2. The injury death rates for rural males were higher than for urban males for the 0-14 and over 35 age groups. There was little difference between urban and rural injury death rates among males aged 15-39 years. Injury death rates for males > 65 years were much higher in rural areas than in urban areas. A somewhat similar pattern was seen for females, with a dramatic increase in the rates among the elderly.

**Figure 3:**
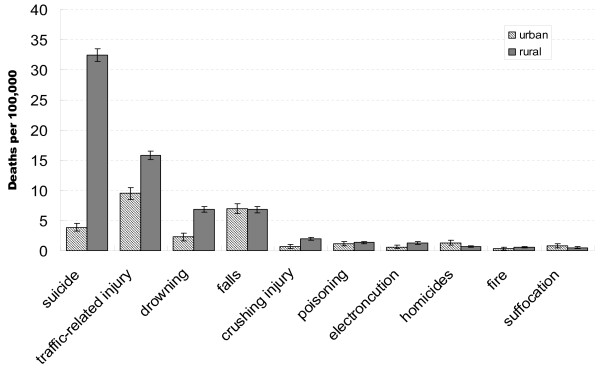
**Age-adjusted injury death rates by cause for urban and rural areas in Hubei Province, China, 2006-2008**.

Injury death rates by external cause for rural and urban residents are summarized in Figure 3. The leading causes of injury death for rural residents were suicide (32.4 per 100 000, 95% CI: 31.4-33.5 per 100 000), followed by traffic-related deaths (15.8 per 100 000, 95% CI: 15.1- 16.5 per 100 000), drowning (6.9 per 100 000, 95% CI: 6.4-7.4 per 100 000), falls (6.8 per 100 000, 95% CI: 6.3-6.8 per 100 000), crushing injury (2.0 per 100 000, 95% CI: 1.8-2.3 per 100 000). In urban area, traffic-related deaths ranked first (9.5 per 100 000, 95% CI: 8.4- 10.5 per 100 000), followed by falls (7.0 per 100 000, 95% CI: 6.1-7.8 per 100 000), suicide (3.9 per 100 000, 95% CI: 3.2-4.5 per 100 000), drowning (2.3 per 100 000, 95% CI: 1.7-2.9 per 100 000), homicides (1.3 per 100 000, 95% CI: 0.9-1.7 per 100 000). The rates in the two regions were similar for falls, poisoning, suffocation and fire. Death rates from suicide, traffic-related injuries, drowning and crushing injuries were significantly higher in rural areas.

**Figure 4:**
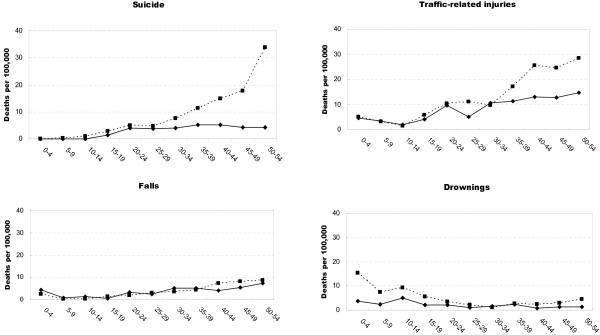
**Age-specific injury death rates per 100 000 by leading mechanism of death for urban (solid line) and rural (dashed line) areas in Hubei Province, China, 2006-2008: 0- 54 year age group**.

**Figure 5:**
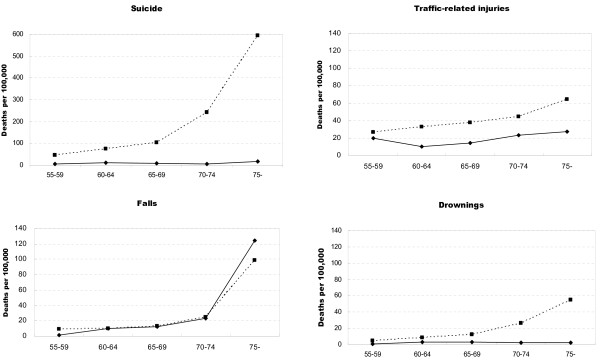
**Age-specific injury death rates per 100 000 by leading mechanism of death for urban (solid line) and rural (dashed line) areas in Hubei Province, China, 2006-2008: ≥55 year age group**.

Age-specific rates for the four leading external causes of death for urban and rural residents are shown in Figure 4 (0-54 year age group) and Figure 5 (≥55 year age group). Suicide rates were significantly higher (relative risk [RR] = 8.5, 95% CI: 6.9-10.4) among adults aged 20 years and over in rural areas than in urban area. Much high rates of suicide were noted in the ≥65 age group in rural areas (279.8 per 100 000, 95% CI: 268.2-291.3 per 100 000) than in urban areas (10.7 per 100 000, 95% CI: 7.1-14.2 per 100 000). The rates for traffic-related deaths were generally similar in rural and urban areas until about age 35 where the rate in rural areas became greater with each older age interval. There was little difference between rural and urban areas for age-specific fall related death rates with a dramatic increase for persons ≥70 years in both areas. Drowning death rates were significantly higher among children aged 0-14 years (relative risk [RR] = 2.8, 95% CI: 1.6-4.6) and persons over 65 years (relative risk [RR] = 3.9, 95% CI: 1.9-7. 9) in rural areas than in urban areas.

## Discussion

Both crude and age-adjusted injury death rates were higher in rural areas than in urban areas in Hubei Province during 2006-2008. Residents of rural areas had an injury death rate twice that in urban areas. Similar findings had been reported elsewhere in China [10,18,19]. Other studies have also identified higher injury mortality rates in rural versus urban locations in most developing and developed countries [20-24]. The urban-rural discrepancy (30.1 vs 70.7 per 100,000) is wider than that of Tanzania (40.5 vs 48.5 per 100,000 in female, 108.8 vs 138.3 per 100,000 in male) [22], Australia (33.2 vs 48.1 per 100,000) [23] and the United states (44.6 vs 31.2 per 100,000) [24].

Surveillance results of injury-related behaviours in Chinese people have indicated that rural residents had more law violations and high-risk behaviours than urban residents [25]. For example, rural residents were more likely than urban residents to drive after drinking in the past month (15% vs 6%), drive without a license in the past month (20% vs 5%), violate pedestrian and bicycle traffic rules in the past 30 days, and store pesticides and rodenticides at home(64% vs 4%). Insufficient emergency medical services and longer response and transport time for trauma care in rural locations may be another factor in higher injury death rates. The third National Health Service survey shows that the density of health care organizations per 10 km2 is 1.41 in urban areas and 0.21 in rural areas, while the density of physicians and nurses per 1,000 population is 3.8 and 3.8, respectively, in urban areas and 1.0 and 0.7, respectively, in rural areas [26].

Rural males had the highest injury death rates in the current study. Likewise, males in rural areas were found to have the highest injury mortality rates in Australia [23], Ireland [20], Canada [21] and Tanzania [27]. In China, rural males have more law violations and high-risk behaviours than urban males [25].

Consistent with earlier studies conducted in China, the suicide death rate in rural areas was higher than the rate in urban areas [28,29]. However in our study the difference is striking. The suicide mortality rate in rural areas in Hubei Province (32.4/100,000) was higher than that of Shandong Province (19.3/100,000) [30], Fujian Province (17.2/100,000) [12] and Hebei Province (7.9/100,000) [11,12]. However, the suicide mortality rate of urban residents in Hubei Province (3.9/100,000) was similar to that of the other three provinces (3.2-6.0/100,000). For those aged greater than 65 years, the suicide death rate among rural residents was 22 times higher than that of urban residents. Comparison of our results with those of other provinces in China and other countries indicates that the rate of suicide among elderly rural residents in Hubei Province was the highest reported for any area.

Most experts believe the frequent use of highly lethal pesticides as a suicide method in rural areas is the main determinant of the higher suicide mortality rate in rural areas [31,32]. However this cannot explain why the suicide rate in rural areas in Hubei Province was higher than the rural rates in other provinces. There are several possible explanations for the wide urban-rural gap in suicide mortality rate in central China, where Hubei Province is located. The urban-rural income gap is larger in central and western China than in the eastern regions [5]. The poorer life and limited welfare provisions and medical support may increase the risk for suicide. The elderly in rural areas tend to receive less financial and emotional support than do their urban counterparts [33]. In recent years, more and more young people have migrated to urban areas to work, and more and more older people in rural areas are living alone [34]. This is especially true in central rural areas, where more young workers have migrated to eastern China [35]. Lack of social support may partly explain the higher rate of suicide in the elderly rural population in central China. In addition, there are no strong religious or legal prohibitions against suicide in China [36], so people with serious mental disorders or chronic life stressors (such as incurable illness) might consider suicide an acceptable method of relieving their misery or of reducing the financial and emotional burden they cause their family. This belief is more popular in rural areas in central China. An empirical study in a county in Hubei province has shown that the widely held conception that old people "should die when they are useless" has created a forgiving, even encouraging, social and psychological milieu for elderly suicide [37].

In contrast to the strong association of suicide with mental illness in the West, China is unique in that it has a low level of mental illness in suicide victims (0-30% vs > 90% in the West), in particular, depression [38,39]. So the western models might not be applicable to China. Economic and physical burdens might be greater factors in suicide among rural elderly in China than elsewhere. Therefore improving social welfare systems for the elderly in rural areas might be effective in combating suicide.

The leading cause of injury deaths in urban areas was traffic-related injury, but mortality rates for traffic-related injuries in rural areas were still higher than in urban areas. Similar results have been shown by studies using national samples [13,19]. This cannot be explained by the difference in use of transportation, which is usually higher in urban regions. Several studies have indicated that the largest proportion of road traffic victims in developing countries are pedestrians, passengers and cyclists as opposed to drivers, in whom most of the deaths and disabilities in the developed world occur [40,41]. Studies conducted in China indicate that 60% of traffic victims are pedestrians, passengers and bicyclists and 20% are motorcyclists [42,43]. The reasons for higher traffic-related mortality in rural communities maybe related to poor-quality roads, less police supervision on the roads, increased presence of vulnerable road users, insufficient emergency medical services, higher rates of driving under the influence of alcohol and higher rates of driving without a license [19,25]. Road sharing by high speed vehicles and walking villagers may also be a contributing factor [44]. Unlike high-income countries in which people aged 15-29 years had the highest death rates of road traffic injury, people 60 years and older had the highest death rates of traffic-related injury in our study which is similar to other low-income and middle-income countries [45]. When involved in a motor vehicle crash, elderly people are more likely to be killed or seriously disabled than younger people because they are generally less resilient [46].

There was little difference between rural and urban areas for fall related death rates. The rate (6.8-7.0 per 100,000) was similar to the worldwide average of 6.6 per 100,000 [47], but lower than that in European regions (6.6-11.3 per 100,000) [47] and India (14.5 per 100,000) [48]. Falls are the second leading cause of fatal injury among urban residents and the third leading cause of fatal injury among rural residents. Similar to other developed and developing countries [47,49], the fall related mortality rate increase with age, with the greatest increase after age 75. The high death rates of falls due to population aging, comorbidities and complicated risk factors in the elderly reflects the need for improvement of the environment and physical conditions of older populations in communities and hospitals in China [50].

The drowning death rate was three times higher in rural areas than in urban areas (6.9 per 100 000 vs 2.3 per 100 000). This finding was consistent with previous studies [11,19,51]. The drowning death rate in our study was higher than that of Hebei Province (3.0 per 100 000 vs 0.8 per 100 000) [11] and was similar to Guangdong Province (6.4 per 100 000 vs 3.7 per 100 000) [51]. There are more waterways in Hubei Province and Guangdong Province. The death rate of drowning in rural areas was similar to that of in South Eastern Asian countries (6.1 per 100,000) while the rate in urban was similar to that of in American (2.7 per 100,000) and other developed countries [47].

Most drownings occurred in the elderly aged 65 years and over and children younger than 15 years in rural areas. Similar to India and Bangladesh [52,53], drownings are the leading cause of fatal injury among children under five years of age and drowning deaths were more common in rural areas amongst these children. The higher drowning death rate among children in rural areas may be related to more children swimming in natural waters or playing in or around natural waters without sufficient supervision [54,55]. The reasons for the higher drowning death rates in the rural elderly population are unclear. One potential explanation may be the misclassification of suicides. There are no coroners' reports for unnatural or accidental deaths in China, so there is an opportunity for family members to influence the physician's recorded cause of death. In some parts of rural China, beliefs in the evil effects of the "wandering spirits" of people who died by suicide might make families reluctant to admit that the death was a suicide [56]. In addition to ingestion of agricultural chemicals or rat poison, drowning in rivers or wells is a common method of suicide in rural elderly [39,57]. We assume that some suicides may be misclassified as drownings.

Limitations of this study include the incompleteness of Disease Surveillance Points (DSP) system data, which may underestimate the true injury mortality and affect our results. A study by the DSP system estimate overall rates of unreported deaths of 13% in rural areas and 15% in urban areas [58]. Another problem in using the DSP data to project death rates by cause is misclassification. About 50% deaths in urban areas and 80% deaths in rural areas were reported through verbal autopsy interviews. Although most injury-related deaths have a defined sequence of events that is less likely to be misclassified, verbal autopsy does not perform adequately for several other causes, including drowning and falls. These injuries cannot be easily differentiated from deaths from other causes. Falls in the elderly may be misclassified as other causes due to co-morbidities, but compensating patterns of misclassification (e.g. classifying other death causes, i.e. stroke, hypertensive diseases, IHD as falls) would appear to suggest that the method yields population-level cause-specific estimates that are reasonably reliable [15,16]. Secondly, our study is limited by lack of information on potential influencing factors. It is based on routinely collected death certificate data, which does not contain other relevant information such as socio-economic status, lifestyle risk factors, occupational history, etc. Therefore it prevents us from understanding the cause of urban-rural difference in injury mortality.

Despite these limitations, the current findings confirm that rural residents are at increased risk for fatal injuries and that these injuries impose a disproportionate burden on rural populations. The most notable disparities were in the death rates for suicide and traffic-related injuries. Additionally, elderly residing in rural locales were at especially high risk for death from injury. Injury prevention strategies and practical actions should be promoted to narrow the gap between urban and rural rates. These include methods of providing economic and mental support to the elderly, improving emergency medical services, restricting the availability of pesticide by ensuring supplies are kept in secure facilities, improving road design to ensure road user safety, constructing safe places to swim and enhancing child supervision. Unfortunately, interventions for reducing high rates of fatal injuries in rural areas are lacking in China. Further research is needed to identify factors leading to high injury deaths rates and to evaluate prevention strategies for reducing injuries and narrowing the rural-urban gap in injury mortality.

## Conclusions

This article has quantified the difference in the experience of injury mortality between rural and urban residents in Hubei Province, central China, with rural residents experiencing a higher injury burden. Suicide, traffic-related injury and drowning that have demonstrably higher mortality in rural compared with urban locations should be targeted for injury prevention activity. There is a need for injury prevention policies targeted at elderly residents, especially with regard to suicide prevention in rural areas in Central China. Our study has provided important information to develop policies and programs that can deliver effective measures in the high risk populations and to set priorities for cause-specific prevention strategies in central China.

## Competing interests

The authors declare that they have no competing interests.

## Authors' contributions

QZ and YG designed the study. LZ, DZ, JL and QZ oversaw data collection. Analysis was undertaken by JL, DK, XS and DZ under supervision of GY. QL drafted the manuscript. All authors contributed to the interpretation of the data and have approved the final version. QZ and YG is the guarantor for the study. All authors read and approved the final manuscript

## Pre-publication history

The pre-publication history for this paper can be accessed here:

http://www.biomedcentral.com/1471-2458/12/180/prepub
